# Identification and Visualization of Functionally Important Domains and Residues in Herpes Simplex Virus Glycoprotein K(gK) Using a Combination of Phylogenetics and Protein Modeling

**DOI:** 10.1038/s41598-019-50490-9

**Published:** 2019-10-10

**Authors:** Paul J. F. Rider, Lyndon M. Coghill, Misagh Naderi, Jeremy M. Brown, Michal Brylinski, Konstantin G. Kousoulas

**Affiliations:** 1 Division of Biotechnology and Molecular Medicine and Department of Pathobiological Sciences, School of Veterinary Medicine, Louisiana State University, Baton Rouge, LA USA; 2 Department of Biological Sciences, Louisiana State University, Baton Rouge, LA USA; 3 Center for Computation & Technology, Louisiana State University, Baton Rouge, LA USA; 4 Museum of Natural Science, Louisiana State University, Baton Rouge, LA USA

**Keywords:** Virology, Herpes virus

## Abstract

Alphaherpesviruses are a subfamily of herpesviruses that include the significant human pathogens herpes simplex viruses (HSV) and varicella zoster virus (VZV). Glycoprotein K (gK), conserved in all alphaherpesviruses, is a multi-membrane spanning virion glycoprotein essential for virus entry into neuronal axons, virion assembly, and pathogenesis. Despite these critical functions, little is known about which gK domains and residues are most important for maintaining these functions across all alphaherpesviruses. Herein, we employed phylogenetic and structural analyses including the use of a novel model for evolutionary rate variation across residues to predict conserved gK functional domains. We found marked heterogeneity in the evolutionary rate at the level of both individual residues and domains, presumably as a result of varying selective constraints. To clarify the potential role of conserved sequence features, we predicted the structures of several gK orthologs. Congruent with our phylogenetic analysis, slowly evolving residues were identified at potentially structurally significant positions across domains. We found that using a quantitative measure of amino acid rate variation combined with molecular modeling we were able to identify amino acids predicted to be critical for gK protein structure/function. This analysis yields targets for the design of anti-herpesvirus therapeutic strategies across all alphaherpesvirus species that would be absent from more traditional analyses of conservation.

## Introduction

Herpesviruses are a significant cause of morbidity and mortality in human and animal populations. To date there are greater than 100 species of viruses recognized in the order herpesvirales including 8 human herpesviruses^1–3^. Mammal, bird, and reptile herpesviruses constitute the family *Herpesviridae* which is divided into alpha, beta, or gamma, subfamilies based on their biological properties, and gene content^1,3,4^. There are approximately 40 genes shared among all subfamilies of herpesvirus, referred to as the core herpesvirus genes^5,6^. These genes encode structural components of the viral nucleocapsid, as well as proteins with functions related to aspects of the viral life cycle that are common to all members of the *Herpesviridae*, such as DNA replication. In addition to the core set of herpesvirus genes, there are genes specific to members of each subfamily. These genes are expected to be responsible for the biological properties of each subfamily that distinguish it from other subfamilies^7^.

Alphaherpesviruses are distinguished from the beta and gamma subfamilies by their ability to establish and maintain latency in the central and/or peripheral nervous systems of the host^7^. After the establishment of latency, these viruses will periodically reactivate and spread to sites of innervation causing clinical symptoms of herpesvirus infection. The ability of alphaherpesviruses to enter cells of the nervous system is central to the pathogenesis of infection by these viruses and to date remains poorly understood. One strategy for identifying genes involved in infection of the nervous system is to focus on those genes unique to alphaherpesviruses. While several of these alphaherpesvirus-specific genes have been implicated in neurovirulence, our understanding of how and which of them facilitate entry, establishment and maintenance of latent infection in cells of the nervous system is incomplete.

gK and UL20 are highly conserved among neurotropic alphaherpesviruses. gK and UL20 have been shown to interact and these interactions are important for virus entry, membrane fusion, cytoplasmic virion envelopment and egress^8–19^. Mechanistically, the gK/UL20 protein complex interacts with the viral fusogen gB and this interaction is necessary for gB-mediated membrane fusion during virus entry, as well as virus-induced cell-to-cell fusion and the formation of syncytia^20,21^. HSV-1 gK is expressed on virion envelopes and functions in virion entry^16,22,23^. Specifically, we have shown that a 39 amino acid N-terminal deletion of gK inhibits virus entry into neuronal axons *in vitro* and *in vivo*, but not other cell types (fibroblasts, epithelial cells)^24–26^.

HSV-1 is the cause of oral herpes and is the prototypical alphaherpesvirus^7^. gK and UL20 are both present in the virion envelope^16,22,27^. Previously, we have shown that gK has three extracellular and two intracellular domains separated by four transmembrane domains^9^. Mutations of gK result in viruses with domain-specific defects in entry, assembly, or egress^17,19,28–31^. These data suggest the domains of gK are functionally distinct, with some involved in entry and others in assembly and egress. At present, the mechanism by which gK functions to facilitate membrane fusion during virus entry and spread, as well as cytoplasmic virion envelopment, is not well understood and is the focus of ongoing work.

Since domains of gK are functionally distinct, we hypothesized that they may exhibit variation in evolutionary rate. Such variation is likely driven by several, interacting factors. First, virion assembly, like DNA replication, may be expected to involve primarily conserved viral protein-protein interactions, while entry is partly the result of interactions between viral entry mediators with -host receptor proteins. Viral protein domains on the surface of virions may evolve faster than internal virion proteins^32^. Additional selective pressure may be expected to occur across a virus family interacting with divergent host species, since they may be subject to diversifying selection imposed by co-evolution with hosts^33^. Extracellular domains (1, 3, and 5) are more likely to be involved in entry as well as be exposed to host antibody binding therefore these domains may be expected to evolve more quickly than intracellular domains (2 and 4). Second, some extramembrane domains may exhibit more structural restraints, while others could be less ordered. Elastic network models allow us to estimate the residue fluctuations, or mobility. We might expect that ordered domains with less fluctuation (e.g., domains 1 and 2; see below) should evolve more slowly than those with less well-defined structures and high fluctuation (e.g., domain 4). Lastly, transmembrane domains need to exhibit a stable core that maintains the integrity of a helical bundle, while residues on the sides of the helical bundle need to remain hydrophobic due to their interactions with membrane phospholipids. Transmembrane domains may also interact with other viral proteins (e.g., UL20)^13,34,35^. Given these constraints, they are generally expected to evolve slowly.

The various factors outlined above may result in contrasting predictions about a domain’s expected evolutionary rate. For instance, an intracellular domain of gK is likely to be involved in virion assembly. Given this role, the domain should have experienced a relatively stable set of interactions during the diversification of alphaherpesviruses, and would be expected to evolve slowly in order to maintain the fidelity of these interactions. However, structural predictions may suggest that the same domain also has a less well-defined structure than other parts of gK, requiring less precision in folding to maintain function. In this case, the domain’s primary sequence may evolve relatively quickly. The contrasts between these predictions allow us to elucidate the relative strength of the predictions.

Overall, our integrated phylogenetic and structural analyses provide new insights in the location and potential functions of gK protein domains. This approach can be extended to the prediction of functional domains and residues of other proteins and glycoproteins, including domains that can be potential targets for antiviral therapy for multiple evolutionary related pathogens.

## Results

### Phylogenetic analysis and the relative evolutionary rate of individual gK domains

**Figure 1:**
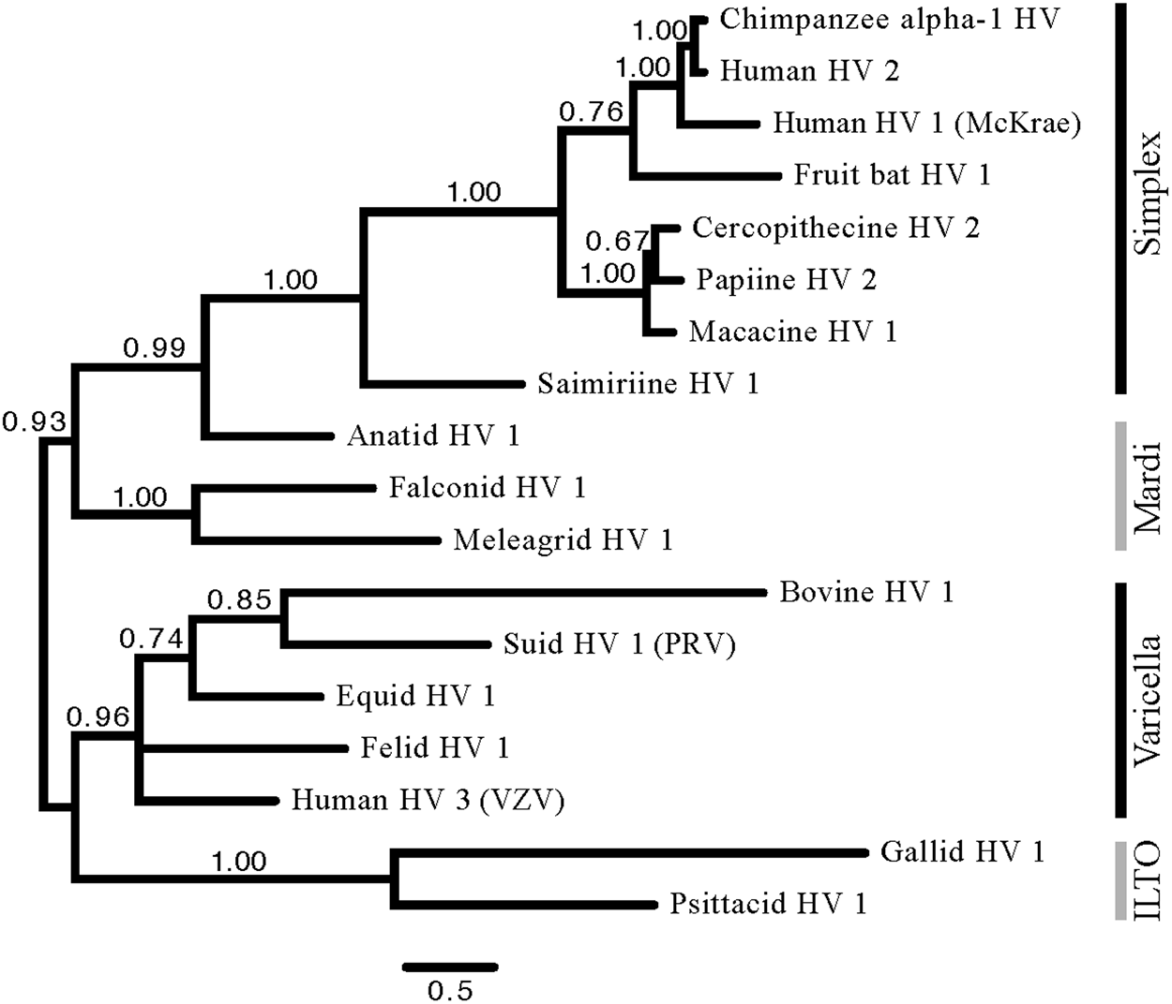
Phylogenetic analysis of alphaherpesvirus glycoprotein K. Phylogeny inferred from Bayesian phylogenetic analysis of gK orthologs from 18 alphaherpesviruses, arbitrarily rooted. Branches are labeled with posterior probabilities. Branch lengths are given as the expected number of substitutions.

We inferred the phylogenetic relationships among 18 species of alphaherpesviruses using a set of genes orthologous to HSV-1 gK (Table 1, Fig. 1). These analyses employed a model of rate variation with a fixed partitioning scheme, including a separate rate multiplier for each gK domain. The topology of the inferred tree is similar to previously published relationships based on whole genome sequences or gene content comparisons^5,36,37^.

**Table 1:** Alphaherpesvirus gK orthologs analyzed in this study.

gK Sequence	Accession Number	% Identity with HSV1
Anatid herpesvirus 1	YP_003084366.1	49.79
**Bovine herpesvirus 1**	**NP_045305.1**	**49.58**
**Cercopithecine herpesvirus 2**	**YP_164497.1**	**71.75**
**Chimpanzee alpha-1 herpesvirus**	**YP_009011041.1**	**89.12**
**Equid herpesvirus 1**	**YP_053051.1**	**50.20**
Falconid herpesvirus 1*	YP_009046554.1	48.47
**Felid herpesvirus 1**	**YP_003331525.1**	**53.34**
**Fruit bat alphaherpesvirus 1**	**YP_009042116.1**	**76.56**
Gallid herpesvirus 1	YP_182337.1	32.42
**Human herpesvirus 2**	**YP_009137206.1**	**88.49**
Human herpesvirus 3 (VZV)	NP_040128.1	46.65
Macacine herpesvirus 1	AIA09548.1	72.17
Meleagrid herpesvirus 1	NP_073348.1	45.81
Papiine herpesvirus 2	YP_443901.1	70.92
Psittacid herpesvirus 1	NP_944381.1	42.05
Saimiriine herpesvirus 1	YP_003933787.1	56.27
Suid herpesvirus 1 (PRV)	YP_068321.1	39.53
**Human herpesvirus 1 (McKrae)**	**AFP86418**	**100.00**
Average		57.85

Species in bold were used for genome-wide phylogenetic analyses. *Presently not recognized as a formal virus species.

**Figure 2:**
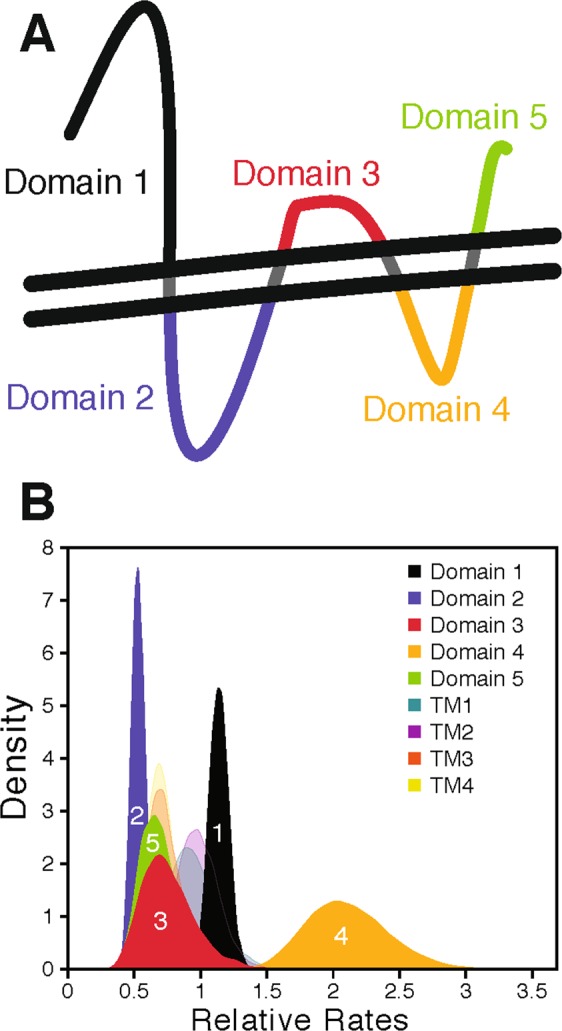
Relative rate of change for individual domains of alphaherpesvirus gK. (**A**) A cartoon representation of gK’s topology with individual domains labeled. The two parallel horizontal lines denote cell membrane. (**B**) Posterior distributions of relative substitution rates for each domain in gK. “TM” indicates a transmembrane domain, which are numbered in the same direction as extramembrane domains. Colors corresponding to extramembrane domains illustrated in A.

To test the hypothesis that domains vary substantially in evolutionary rate based on function, we inferred these rates for each of the 5 extramembrane and four transmembrane domains (Fig. 2A) across the 18 gK orthologs listed in Table 1. To determine the locations of the transmembrane domains for the HSV-1 gK orthologs, sequences were submitted to tmfoldweb^9^. With one exception, all gKs were predicted by tmfoldweb to possess 4 transmembrane domains and five extramembrane domains with a similar structure to HSV-1 gK. The only exception was PRV gK, which was predicted to have 6 extramembrane domains and 5 transmembrane domains. This alternative prediction was due to the separation of domain 2 into two separate domains (one cytoplasmic and one extracellular). Since all 17 other gK sequences were predicted to possess the same topology as HSV-1, we combined the two domains predicted for PRV domain 2, yielding five extramembrane domains across all orthologs.

We found that domain 2 had the lowest inferred rate of evolution, while domain 4 had a substantially faster rate than all others (2–4 fold faster; Table 2, Fig. 2B), despite both being intracellular. The extracellular domains (1, 3, and 5) had intermediate inferred rates, with domain 1 evolving faster than domains 3 and 5. The transmembrane domains also had intermediate rates, roughly spanning the same range as the extracellular domains. Unsurprisingly, domains with more residues (e.g., 1 and 2) produced more precise estimates of rate (Fig. 2B).

**Table 2:** Relative rates of change for gK domains. A value of 1 is the average rate across all gK residues.

	Domain 1	Domain 2	Domain 3	Domain 4	Domain 5	TM1	TM2	TM3	TM4
mean	1.1877	0.3377	0.6368	2.3745	0.4787	0.6938	1.0055	0.8736	0.7968
stdev	0.0953	0.0529	0.2429	0.3909	0.1509	0.1747	0.2047	0.2091	0.1661

### Analysis of the relative evolutionary rate of glycoprotein K (gK)

**Figure 3:**
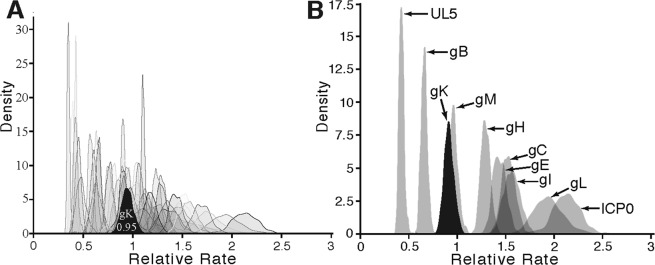
Rate of change for alphaherpesvirus gK relative to conserved alphaherpesvirus ORFs. (**A**) The posterior distribution of gK’s relative substitution rate compared to those from 58 other alphaherpesvirus genes. (**B**) Posterior distributions of relative substitution rates for a subset of genes found in (**A**), focusing on glycoproteins. UL5 (slowest) and ICP0 (fastest) are also included as references.

We created a concatenated alignment of 59 orthologs present in all alphaherpes viruses and employed a partitioned model with independent rate multipliers for each gene, allowing us to compare the rate of evolution of gK relative to the rest of the genome (Fig. 3A). To improve mixing and convergence of these Markov chain Monte Carlo (MCMC) analyses, we fixed the tree topology using the majority-rule consensus from the gK results. The estimated rate multiplier for gK was 0.95, indicating that it is evolving at a rate roughly average across the 59 orthologs. Other orthologs varied substantially in their estimated rates, with multipliers ranging from roughly 0.4 to greater than 2 (Fig. 3a). This range roughly parallels the range of estimated rates across domains within gK.

Focusing only on the shared glycoproteins, gK has a rate that is second lowest after glycoprotein B, the major mediator of herpesvirus fusion (Fig. 3B). This relationship between the evolutionary rates for glycoproteins was previously demonstrated in an analysis restricted to HSV-1 and HSV-2^38^ and holds true for comparisons among all alphaherpesvirus species in our analysis. For comparison, we included ICP0 and UL5 in these comparisons, although they are not glycoproteins. ICP0 and UL5 are known to have high and low evolutionary rates, respectively. In agreement with previous results^39^, ICP0 had a higher evolutionary rate than all glycoproteins (Fig. 3B) and UL5 exhibited the lowest rate of change. ICP0 is ascribed multiple, disparate functions including E3 ubiquitin ligase and transactivation activities^7^. UL5 is involved in DNA replication, a function that is expected to exhibit a high degree of conservation regardless of host variation^7^.

### Analysis of the relative evolutionary rate of individual gK amino acids

**Figure 4:**
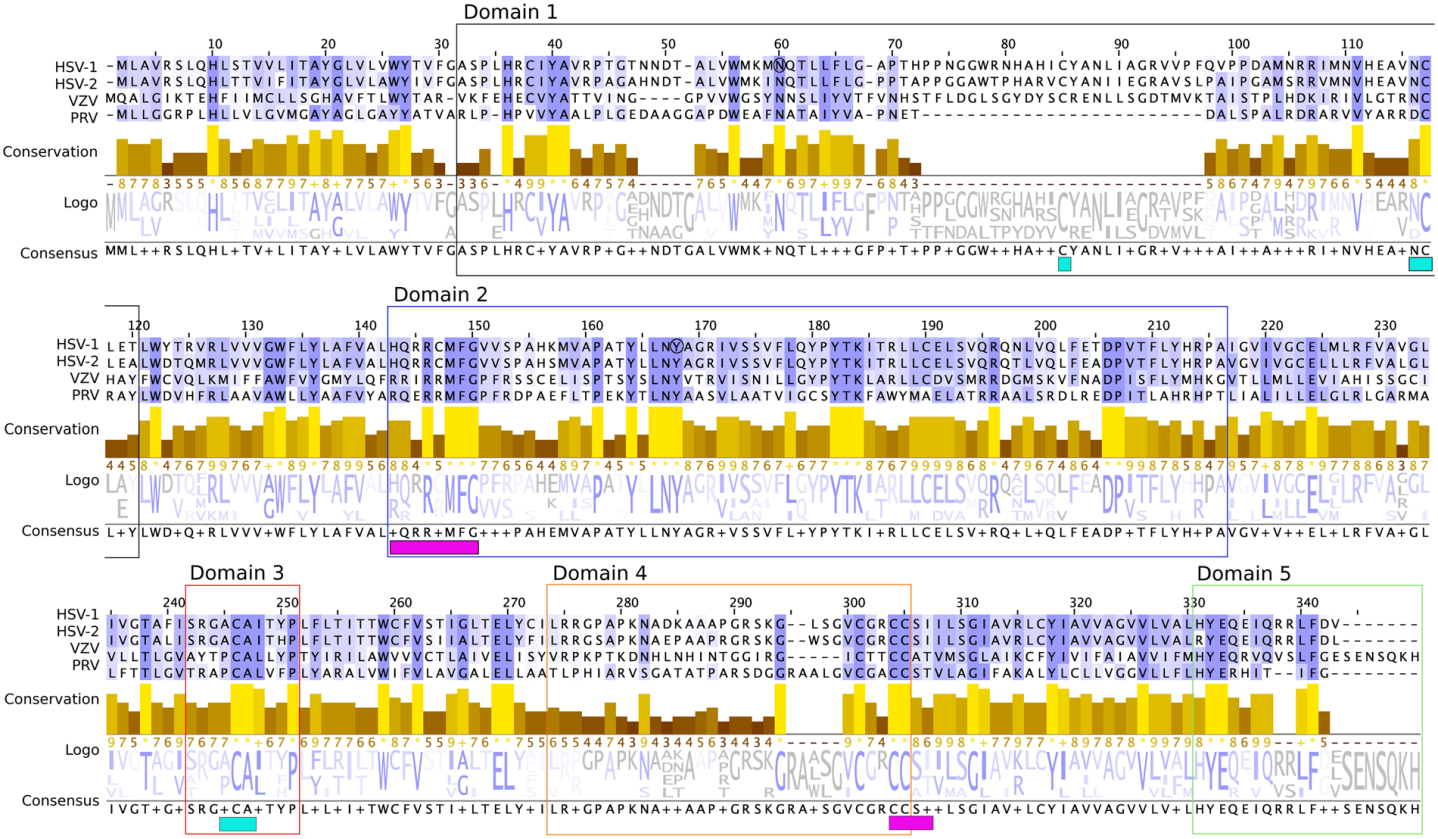
Multiple sequence alignment of selected herpes simplex and varicella gKs. Amino acid sequences for gK are from HSV-1, HSV-2, VZV, and PRV. Residues are color coded according to Jalview BLOSUM62 color scheme. The consensus sequence represents the modal residue at each column and “+” signifies where the modal residue is ambiguous. Conservation scores are shown as histograms and indicate the degree of preservation of physicochemical properties for each residue. The highest score of 11 (depicted in the lightest shades and indicated with “*” below the histogram) shows fully conserved residues, while values close to 11 indicate some change in amino acid identity while maintaining physicochemical properties. Extramembrane domains are boxed and colored according to graphical representation of domains shown in Fig. 3. Magenta (Domains 2 and 4) and cyan (Domains 1 and 3) bars at the bottom represent amino acid clusters that are brought together in the folded gK structure featured in Fig. 6, circled by the same colors.

To illustrate sequence features of gK, orthologs from simplex virus genera (HSV-1, HSV-2) and varicellovirus genera (VZV and PRV) were aligned using clustal omega^40,41^ (Fig. 4). Two important features can be seen in this alignment. First, all four of these gK sequences possess two putative N-linked glycosylation sites separated by 9–10 amino acids in domain 1. These sites occur at positions 49 and 60 in the alignment (Fig. 4) for the simplex viruses and positions 60 and 70 for the varicelloviruses. Second, domain 2, which has the lowest rate of change for all gK domains (Fig. 2), possesses multiple conserved tyrosine residues suggesting this domain may be involved in protein-protein interactions that facilitate assembly.

To develop a comprehensive and quantitative understanding of variation in evolutionary rate across individual residues, including identification of those that may be under strong constraints, we employed a novel finite mixture model (FMM) for among-site rate variation. While site rate estimates did vary somewhat as the number of mixture components changed (Figs S1–3), the rates were quite consistent across analyses using either 4 or 6 components (Fig. S3). Individual sites exhibited more than 20-fold differences in inferred rate, with a strong mode at intermediate rates (Fig. S4; tree length ≈ 12). The overall distribution of site rates was roughly symmetric, but included smaller modes at slower (tree length ≈ 6) and faster (tree length ≈ 17) rates.

### Structural prediction of alphaherpesvirus gKs

**Figure 5:**
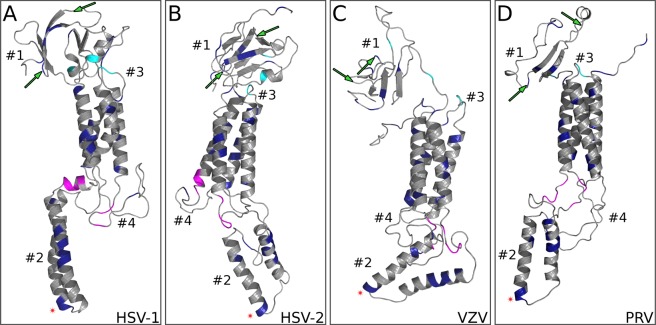
Structural predictions of selected simplex and varicella gKs. Cartoon representations of simplex (HSV-1, HSV-2), and Varicella (VZV, PRV) gK protein models are shown in (*A)*-(*D)*. Residues conserved across all four sequences (Fig. 4) are shown in color. Magenta and Cyan colors correspond to the alignment (Fig. 4). Predicted N-glycosylation sites are marked by green arrows. Asterisk indicates YTK sequence in domain 2.

Structure based analyses can reveal protein features not evident from amino acid sequence alone^6^. These analyses are particularly valuable for comparison of gKs from the simplex and varicella genera of alphaherpesviruses that possess little amino acid sequence identity, but which are expected to have functional conservation. Additionally, gK has extensive hydrophobic regions that preclude the use of traditional methods to study structure, such as crystallization and circular dichroism. To determine the degree of structural conservation between representative varicello and simplex herpesvirus, we predicted the structures of HSV-2, VZV and PRV and compared these to our previously published HSV-1^42^ predicted gK structure (Fig. 5). We found that all four alphaherpesvirus gKs were structurally congruent for domains that possess a discernable structure (domains 1 and 2). We have shown that mutations in domain 1of gK render the virus incapable of entry into cells via neuronal axons^26,42^. Interestingly, the domain with the highest rate of change (domain 4) is also predicted to be the least ordered (Fig. 5).

**Figure 6:**
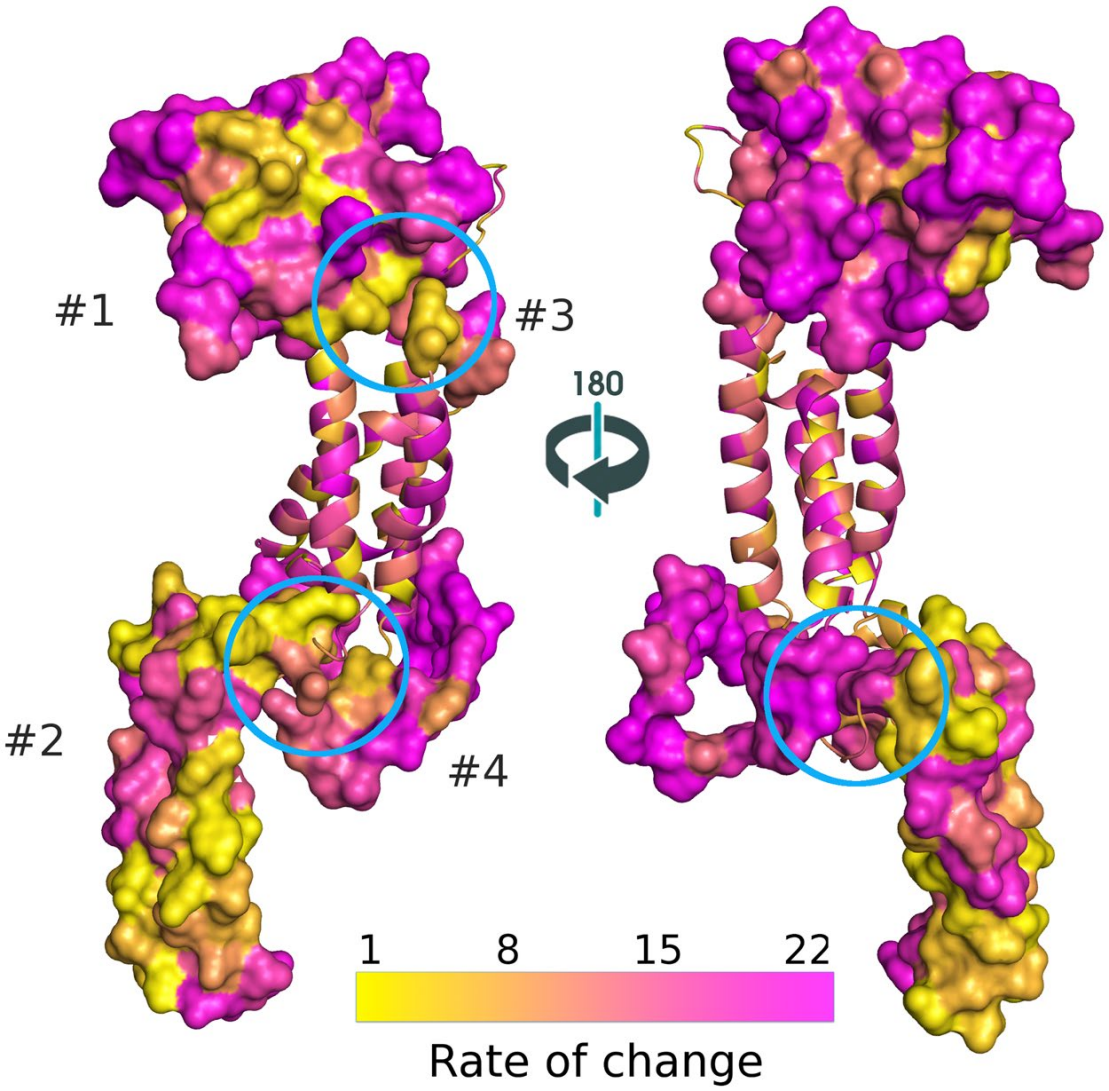
Site-specific relative rates of change mapped onto the predicted structure of HSV-1 gK. Relative rates of change for each amino acid in HSV-1 gK are mapped onto the predicted structure. Transmembrane and extramembrane domains are shown in ribbon and surface representations, respectively. Domains 1 and 3 proximal residues are marked with a cyan circle and proximal residues between domains 2 and 4 are marked with a magenta circle, corresponding to the alignment (Fig. 4). Purple and gold color scheme indicates high and low relative rate of change, respectively.

To visualize conserved amino acids on the structure of HSV-1 gK, we generated a heat map with each amino acid colored according to its relative rate of change (Fig. 6). Overall, one can clearly see that spatially clustered sites often have similar rates, despite the fact that the model of sequence evolution we employed assumes that each site evolves independently. Figure 6 also shows the conservation of amino acids at the predicted interface between domains 1 and 3, as well as domains 2 and 4. Interestingly, the conserved, slow evolving residues of domain 2 form an almost mirror image across two predicted alpha helices that extend into the cytoplasm. Additionally, we found that the position of the YTK sequence of domain 2 is conserved at the tip of the first alpha helix in all alphaherpesvirus examined. We previously showed that this tyrosine is functionally important, as a virus with a Y183S mutation was severely attenuated *in vitro*^17^.

## Discussion

Alphaherpesviruses are significant human and animal pathogens, creating a substantial burden of morbidity and mortality across many species. They have also been subject to diverse evolutionary pressures, given their expansive host range. Future therapeutic efforts to prevent or control alphaherpesvirus infection will be most effective if they exploit viral characteristics that are evolutionarily conserved across the entire group and critical to their function. In this study, we employed a combination of phylogenetic and structural analyses to understand how the viral glycoprotein gK, which is required for virus entry into neuronal axons, has evolved across this diverse group of viruses and to predict which of its domains and residues are most important for maintaining critical functions.

We found that while glycoprotein K is highly conserved across all alphaherpesviruses, and it is known to perform critical roles in neuronal infection^25,26,42^, its rate of sequence evolution is not remarkable. Specifically, gK evolves at a rate roughly average across the herpes viral genomes examined. In contrast to its overall sequence evolution the predicted structure of gK for specific domains was predicted to be more highly conserved. The disconnect between relative rate of sequence evolution and structural conservation for gK may result from the need to adapt to diverse hosts while maintaining structural similarity. In support of this hypothesis, our combined analysis suggests that, while gK lacks correlation between structure and sequence evolution across its entirety, the correlation between protein domains with low rates of sequence evolution possess greater structural conservation.

Extramembrane domains of gKs vary more in evolutionary rate than transmembrane domains, but their rate is not well predicted by their position in the protein nor their presumed function. Instead, the residue fluctuation (estimated Root Mean Square Factor (RMSF)) and the degree of ordering in their structures (Figs S5, 6) appears to be a more important predictor of a domain’s average rate of evolution. Transmembrane domains (lighter colors in Fig. 3B) evolved at relatively similar rates, consistent with the expectation that they must maintain general structural features (e.g., stable helices with hydrophobic exteriors), but have flexibility in the precise identities of many amino acids. Interestingly, cytoplasmic domains (2 and 4) did not evolve consistently faster or slower than extracellular/lumenal domains (1, 3, and 5). Instead domain 2 had the slowest relative rate, while domain 4 had the highest rate. This ranking suggests that the degree of ordering in a domain’s structure is a more important factor in determining its rate of evolution than its probable function. The slow average rate for domain 2 also suggested that it harbored many sites constrained by selection, and that alteration of these residues may disrupt protein function more than similar changes in other domains.

Site-by-site variation in evolutionary rate was inferred to be 10-fold greater than variation in average rate across domains and genes (Figs 2, 3, 6, and S4). This result is not surprising, since averages are by definition intermediate, but it reinforces the need to examine site-specific properties in order to predict functional or evolutionary significance. Critical functions of proteins are likely to be controlled by a subset of residues, whose properties may easily be overlooked when averaged across surrounding sites. In turn, the importance of inferring site-specific properties highlights the need to expand the available set of sequence evolution models. Here, we applied a novel finite mixture model (FMM) to infer site-specific rates. This model formulation has several advantages, including minimal assumptions about the distribution of rates across sites and a parameter space that allows for efficient estimation of the posterior distribution. While our FMM is less flexible than the Dirichlet process prior model (DPP) model proposed by Huelsenbeck and Suchard (2007), our limited tests suggest that it is easier to apply and achieved stable rate estimates with a small number of categories (Fig. S3).

Despite being structurally naive, the model of sequence evolution that we applied resulted in site-specific rate estimates that are spatially clustered (Fig. 6) and often make sense in the context of a predicted functional role. For instance, despite the overall high rate of evolution inferred for domain 4, residues that are predicted to interact with domain 2 have very low site-specific rates (magenta circle in Fig. 6). The same is true for residues that interact between domains 1 and 3 (cyan circle in Fig. 6). While site-specific rate estimates can often be very informative, they also exhibit considerable uncertainty. By placing these estimates in the context of a predicted protein structure, we gain greater confidence that sites with low predicted rates are functionally important and worth exploring experimentally. Further, the rate estimates, by being structurally naïve support features of our structural models such as the potential interaction between domains.

A general region of potential functional importance was domain 2 of gK, given its overall slow rate. In structural predictions for HSV-2, VZV, and PRV, domain 2 exhibits a highly conserved double alpha helix that extends into the cytoplasm. This structure is consistent with its presumed role in virus assembly, a process conserved across alphaherpesviruses. Of specific interest are a number of conserved tyrosines within gK domain 2. Our molecular model and phylogenetic analyses indicate that these tyrosines may play important roles in how gK regulates gB-mediated membrane fusion. Similarly, cytoplasmic virion maturation is a coordinated process that is both spatially and temporally regulated^7,43^. These tyrosines may be involved in protein-protein interactions and/or the appropriate localization of gK to facilitate cytoplasmic virion envelopment. In this regard, we recently showed that the HSV-1 UL37 tegument protein interacts with gK via specific tyrosine residues located within conserved UL37 regions^35^.

While less conserved overall, other domains also contained individual residues predicted to be of functional importance. Domain 3 possesses remarkable conservation of a central cysteine residue across all alphaherpesvirus examined suggesting that this domain plays an important role in infectious virus production. Similarly, there are 3 conserved cysteines in the extracellular domain 1, which may be involved in conserved disulfide bonding between domains 1 and 2. We previously showed that mutation of the conserved C304S-307S in domain 4 resulted in a virus with attenuated growth^17^. Therefore, while domain 4 had the highest rate of evolution, it may still possess a conserved function. Conversely, the slow rate of evolution for domain 5 is surprising, since we previously showed that deleting this entire domain has little effect on viral replication *in vitro*^17^. Although deletion of domain 5 does not adversely affect virus replication *in vitro* domain 5 may be important for gK function *in vivo*. Transmembrane and near-transmembrane regions may also have conserved features to facilitate proper localization or protein-protein interactions. For instance, in our template based models of alphaherpesvirus gKs, residues near the ends of transmembrane helices are often conserved (Fig. 4), possibly due to interactions with different phospholipid composition on the two sides of the two membrane leaflets. Additionally, every helix in the transmembrane bundle exhibits a side with more stable residues towards the inside of the bundle and a more mutable side towards the membrane (Fig. 6), although we have not formally analyzed this pattern. The same general theme can be seen in Fig. 4, where more conserved residues are located in the transmembrane domains at almost every four amino acids.

In this work we focused on the identification of conserved regions in alphaherpesvirus gK. However, functionally important regions of divergence are likely to be involved in host-range specificity including virus entry, spread and overall *in vivo* pathogenicity We have shown in previous work that the number of amino acids between N-linked glycosylation sites within gK domain 1 are correlated with host range^23^. For example distances of 11 amino acids are found in primate and non-human primate herpesviruses whereas distances of 8 amino acids are found in most avian herpesviruses that were examined. Additional work from our laboratory has demonstrated that mutations in gK domain 1 modify receptor specificity of HSV-1^44^. Finally, while simplex viruses infect and replicate in the epithelium before entering enervating neurons via their axonal termini other viruses have differing biology of infection. For example VZV is hematogenously disseminated after transfer to T-cells. After dissemination VZV infects neurons via the axonal termini or cell body^45^. In addition VZV lacks the receptor binding member of the receptor complex, gD, and is highly cell associated with the generation of very few free virus particles^46^. Using data generated in this paper, a future focus of research will be to test the function of both conserved and divergent residues of gK across multiple families of alphaherpesvirus species.

Our approach of combining evolutionary and structural analyses to predict functional sites is readily applicable to the study of other proteins and will likely complement standard approaches to understanding their function. While we have focused mostly on similarities across alphaherpesvirus gK orthologs, our analyses also identify areas of rapid evolution that may contribute to differences in infection and pathogenesis across species. Domain 1, in particular, is known to control cellular receptor utilization by the virus and has likely been the target of diverse selection pressures. The same approach that we have employed here could be used to investigate host-specific adaptation in the future. Overall, these analyses contribute to a greater understanding of both the evolution and structure of an important viral pathogenesis determinant and represent a general approach for the rational design of drugs to target multiple human and animal alphaherpesvirus pathogens.

## Materials and Methods

### Phylogenetic and evolutionary rate analysis

Eight previously sequenced HSV strains were selected from Genbank to be included in genome-wide phylogenetic analyses (bolded species in Table 1). These sequences were chosen to represent all major groups of alphaherpesviruses, while also focusing on those found in humans (HSV-1, HSV-2, and VZV). In order to conduct phylogenetic analyses, homologous regions of the genome must be identified and aligned. We conservatively interpreted available annotations to identify homologous regions, resulting in 59 homologous gene products that were extracted from each genome and compiled into gene-by-gene (amino acids) datasets. Each gene-by-gene dataset was aligned with MAFFT version 7 using the E-INS-i algorithm for the highest level of accuracy^47^. In order to select the best fitting model of amino acid substitution, we used the Bayesian Information Criterion (BIC) as implemented in prottest version 3.2^48^. All available 120 amino acid substitution models were tested, and in each case the Jones92 model with invariant sites and a gamma distribution was preferred by BIC. All individual gene datasets were then concatenated, resulting in an alignment of approximately 41,543 amino acids from each of 59 genes (a total of ~2.5 million amino acids).

Bayesian analyses with fixed partitioning schemes were performed using MrBayes version 3.2.5^49^. Fixed partitioning requires making a priori decisions about which sites we expect to evolve with similar dynamics or at a similar rate. Similar sites are then assigned to the same model of sequence evolution. Fixed partitioning analyses included both those that estimated parameters independently across 59 genes (to compare the relative rate of gK to other genes), and those that estimated domain-specific parameters for gK. All substitution model parameters, with the exceptions of topology and branch lengths, were unlinked across data subsets, including relative rates of evolution for each gene or partition. Each analysis included 4 independent runs of 5 million generations with 4 Metropolis-coupled chains per run. Analyses were monitored for convergence by examining trace plots of scalar values in Tracer version 1.6^50^. Trace plots show the values of parameters sampled across MCMC generations. Similarity in these values through time, and across runs, improves confidence that we are accurately estimating the posterior distribution. Topological convergence was assessed using the average standard deviation of split frequencies (ASDSF). All analyses were conducted both with and without missing data (gaps). In all cases, recovered topologies and support values did not exhibit strong conflicts, so we only report results from complete datasets. A majority-rule consensus tree was constructed in MrBayes and plotted using FigTree version 1.4.2 for the gK analyses (Fig. 1)^51^. The relative rates of evolution of different genes or domains were compared using inferred rate multipliers.

For domain specific analyses, eighteen viral amino acid sequences for the envelope glycoprotein K gene (gK) were taken from Genbank (Table 1). These sequences were then aligned by hand, using structure as a guide, which resulted in an alignment with 478 positions. HSV-1 gK is 338 amino acids long and when aligned with other gKs the gaps result in an alignment that is overall 478 positions. This alignment was partitioned into individual domains, also based on structure (Figs 4–6, and Supplementary Material). Model selection was performed as described above.

To estimate the relative rates of evolution for each residue in gK, we employed a novel partitioning model, where the assignment of sites to rate categories is not fixed. This model is closely related to the nonparametric method of Huelsenbeck and Suchard, who considered the number of rate categories to be a random variable distributed according to a Dirichlet process prior model (DPP)^52^. We initially explored the use of the DPP, but found that mixing and convergence were difficult to achieve for our data, resulting in site-specific estimates that were not reliable. Instead, we employed a finite mixture model (FMM), where the number of rate categories (*k*) is fixed *a priori*, although the assignment of sites to categories is random. Following the notation of Huelsenbeck and Suchard^52^ the assignment of sites to categories can be given as a vector of mappings ${\boldsymbol{\sigma }}=({\sigma }_{1},\,\mathrm{...},\,{\sigma }_{n})$, where *n* is the number of sites in the alignment and ${\sigma }_{i}\in \{1,\,\mathrm{...},\,k\}$. We explored two versions of this FMM: one where the prior probability of each site’s assignment to each category is fixed and equal, $P({\sigma }_{i}=1,\,\mathrm{...},\,k)=1/k$, and another where a Dirichlet distribution is employed as a hyperprior on the probabilities of assignment, $P({\sigma }_{i}=1,\,\mathrm{...},\,k) \sim {\rm{Dir}}({\boldsymbol{\alpha }})$. In the latter form, ${\boldsymbol{\alpha }}=({\alpha }_{1},\,\mathrm{...},\,{\alpha }_{k})$, where *α*_*i*_ is the concentration parameter for category *i*. All of our analyses employed a flat Dirichlet with ${\boldsymbol{\alpha }}=(1,\,\mathrm{...},\,1)$. For both versions of the FMM, we explored the use of 2, 4, and 6 discrete rate categories, each with an independent tree length. Replicate analyses were compared to ensure consistency of site-specific rate estimates. The number of categories was increased until rate estimates stabilized as more categories were added (Figs S1–3). Our primary goals were to estimate the rankings and relative magnitudes of site-specific rates, and we found that these estimates were relatively stable between the 4- and 6-category analyses. These analyses were performed with RevBayes v1.0.1^53^ and the associated Rev scripts are available as supplementary material (https://github.com/jembrown/Evolution2017/blob/master/Example_FMM.rb).

### Multiple sequence alignment

Multiple sequence alignment (MSA) between four different strains of alphaherpesviruses HSV-1, HSV-2, PRV, and VZV (UniProtKB accession number P68333, G9I276, Q85230 and Q4JQX0). The MSA was constructed with Clustal Omega sequence profile alignment^54^ by Jalview software^55^ and visualized using the Jalview Blosum62 color scheme; in this color scheme gaps are colored white. If a residue matches the consensus sequence residue at that position it is colored dark blue. If it does not match the consensus residue but the 2 residues have a positive Blosum62 score^56^ it is colored light blue. Conservation and sequence logo analysis alignment is generated and visualized by Jalview.

### Computational protein structure modeling

Template-based modeling of gK proteins from four alphaherpesvirus, HSV-1, HSV-2, PRV, and VZV (UniProtKB accession number P68333, G9I276, Q85230 and Q4JQX0), were achieved by dividing the protein sequences into domains, modeling each domain separately using Modeller^57^. The extramembrane domains were separated by the transmembrane domains. The locations of the transmembrane domains were determined using tmfoldweb server. Secondary protein structures were predicted using PSIPRED and JPRED. Secondary structure elements were enforced in the modeling process. In order to model the transmembrane domain into one alpha helix bundle rather than separate alpha helices, the best template structure was found using tmfoldweb server. The X-ray crystal structures of E. coli YEDX (PDB code: 2igl, chain: D), MOUSE CLAUDIN-19 (PDB code: 3 × 29), Epstein-Barr virus gH and gL complex (PDB code: 3PHF, chain: Y) were used as templates for the N-terminal, the transmembrane and the domain 2 for HSV-1, HSV-2, and VZV. In case of modeling the N-terminal domain for PRV, Pichia Pastoris Lysyl Oxidase (PDB code: 1W7C, chain: A) was used as the template. All of these templates showed high scores with low E-values. Individual domains were assembled according to the current understanding of each domain; the N-term and TM domains were specifically positioned relative to each other by superimposing separate domain structure models onto MOUSE CLAUDIN-19 protein crystal structure (PDB code: 3 × 29). Full-chain protein structures were remodeled using Modeller to refine loops and gaps in between domains.

### In silico alanine scanning

Amino acid residues in the gK homology model are mutated into alanines one at a time by AlaScan feature in the protein design package FoldX Suite downloaded in 2018 (http://foldxsuite.crg.eu/); the difference in the free Gibbs energy change (∆∆G) before and after performing each mutation is calculated by FoldX Suite. The numbers for the specific mutations are reported as deviations from the average; a large positive ∆∆G indicates the importance of an amino acid residue for structure stability.

## Supplementary information

Supplementary Information

## Data Availability

All of the data are fully accessible at the following url: 10.5061/dryad.8g0d43c.
