# Supplementary material for: Identification and Visualization of Functionally Important Domains and Residues in Herpes Simplex Virus Glycoprotein K(gK) Using a Combination of Phylogenetics and Protein Modeling
Source: Sci Rep. 2019 Oct 10;9:14625. doi: 10.1038/s41598-019-50490-9 (PMC6787236; doi:10.1038/s41598-019-50490-9)
Supplement: Supplementary file 1 — Supplementary Information [file 41598_2019_50490_MOESM1_ESM.pdf]

Supplementary Information for:

Identification and Visualization of Functionally Important Domains and Residues in Herpes Simplex Virus Glycoprotein K(gK) Using a Combination of Phylogenetics and Protein Modeling

Paul J.F. Rider[1]*, Lyndon Coghill[2]*, Misagh Naderi[2]*, Michal Brylinski[2], Jeremy M. Brown[2], Konstantin G. Kousoulas[1#]

[1]Division of Biotechnology and Molecular Medicine and Department of Pathobiological Sciences, School of Veterinary Medicine, Louisiana State University, Baton Rouge LA

[2]Division of Biological Sciences, Louisiana State University, Baton Rouge LA

*These authors contributed equally to this work.

#Correspondence: Konstantin G. Kousoulas

Email: vtgusk@lsu.edu

Tel: 225-578-9682

Fax: 225-578-9665

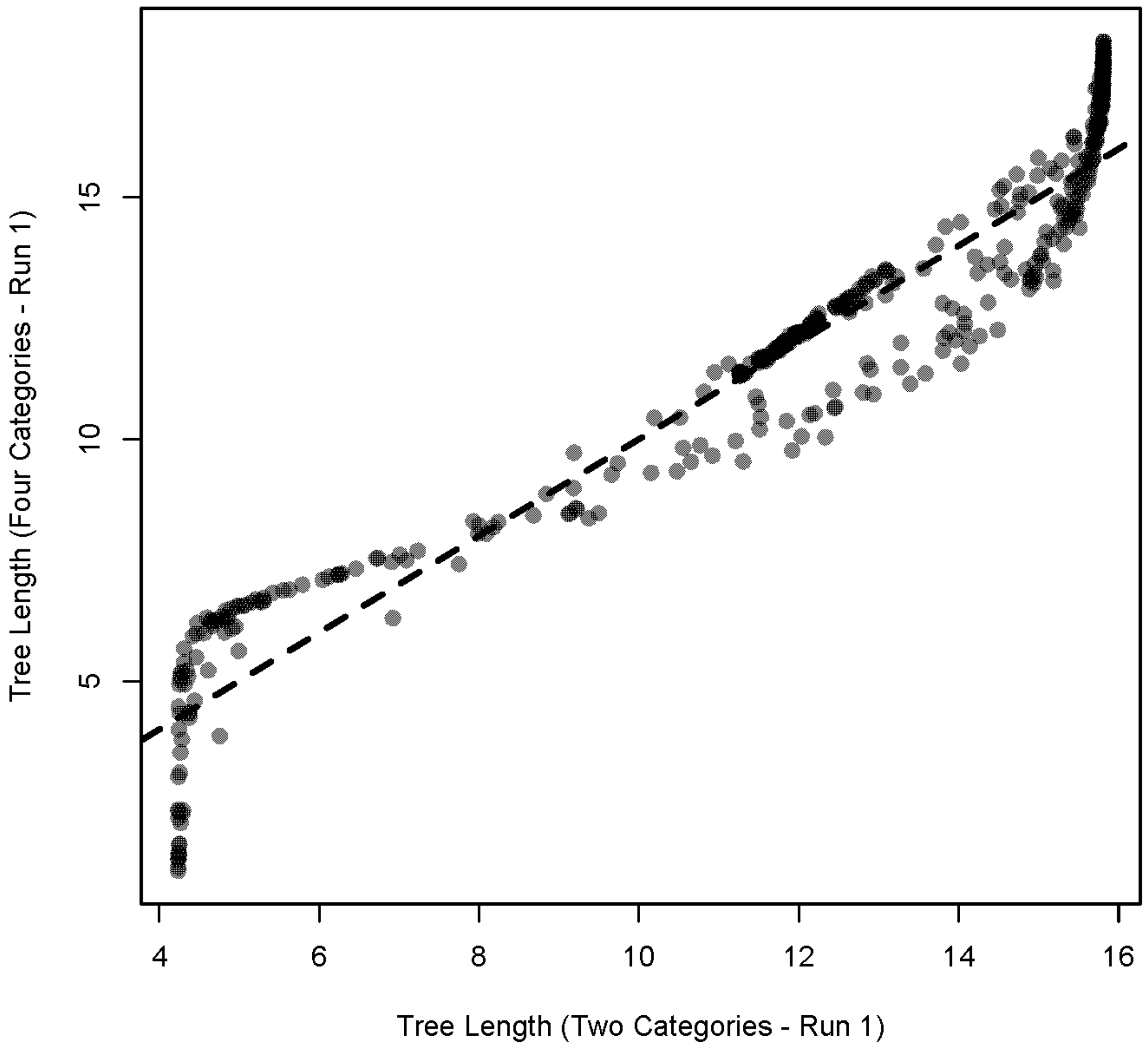

Figure S1. A comparison of site-rate (tree length) estimates between finite mixture models (FMMs) with two and four categories. The number of categories is the number of unique site rates across the protein. For instance, in a two-category model, all sites have one of two possible rates. The values of these rates are estimated from the data. Each point represents one site in the alignment of all gK domains. The mean posterior tree length from the two-category FMM is given on the *x*-axis and the corresponding value from the four-category FMM is given on the *y*-axis.

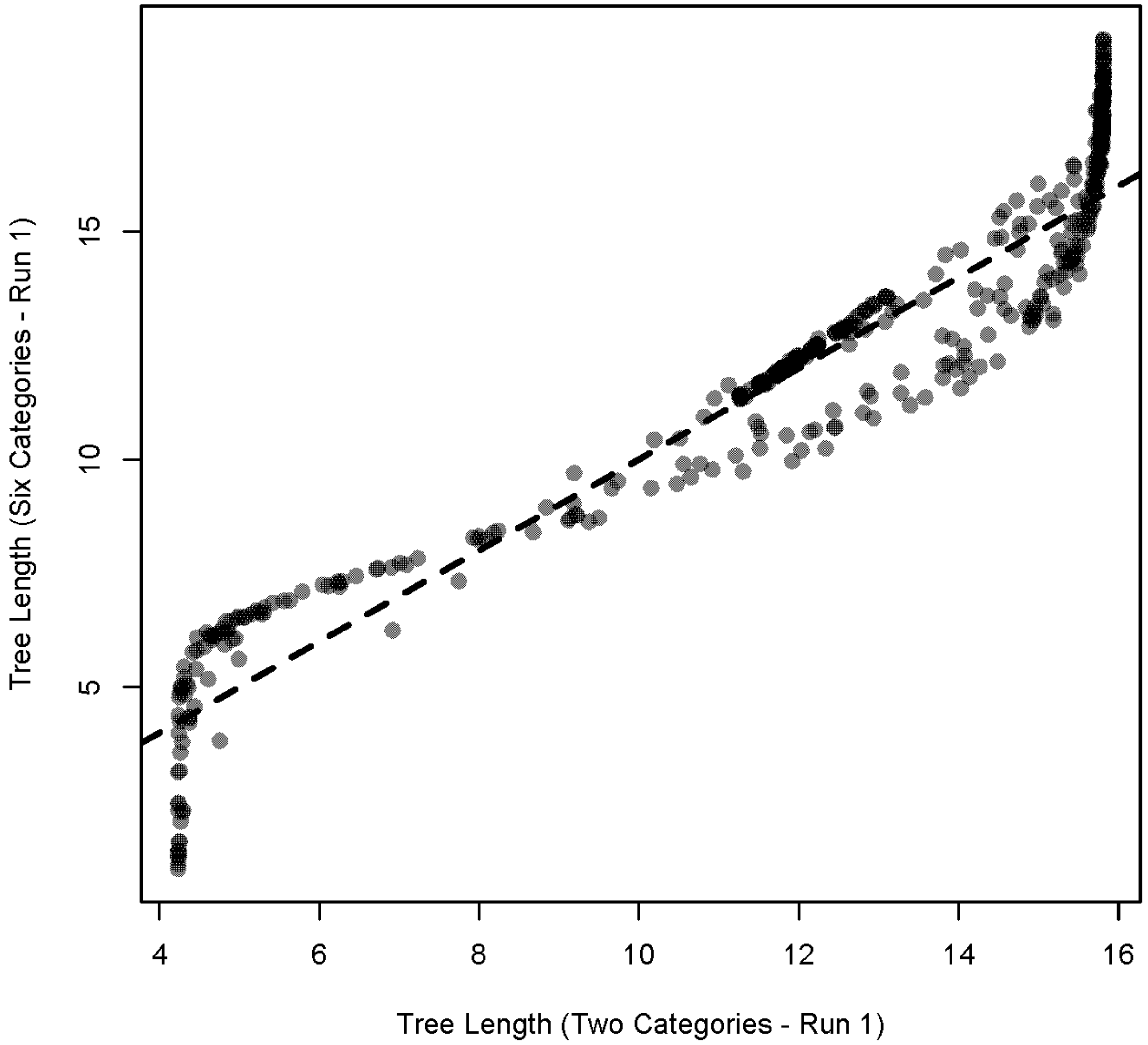

Figure S2. A comparison of site-rate (tree length) estimates between FMMs with two and six categories. Each point represents one site in the alignment of all gK domains. The mean posterior tree length from the two-category FMM is given on the *x*-axis and the corresponding value from the six-category FMM is given on the *y*-axis.

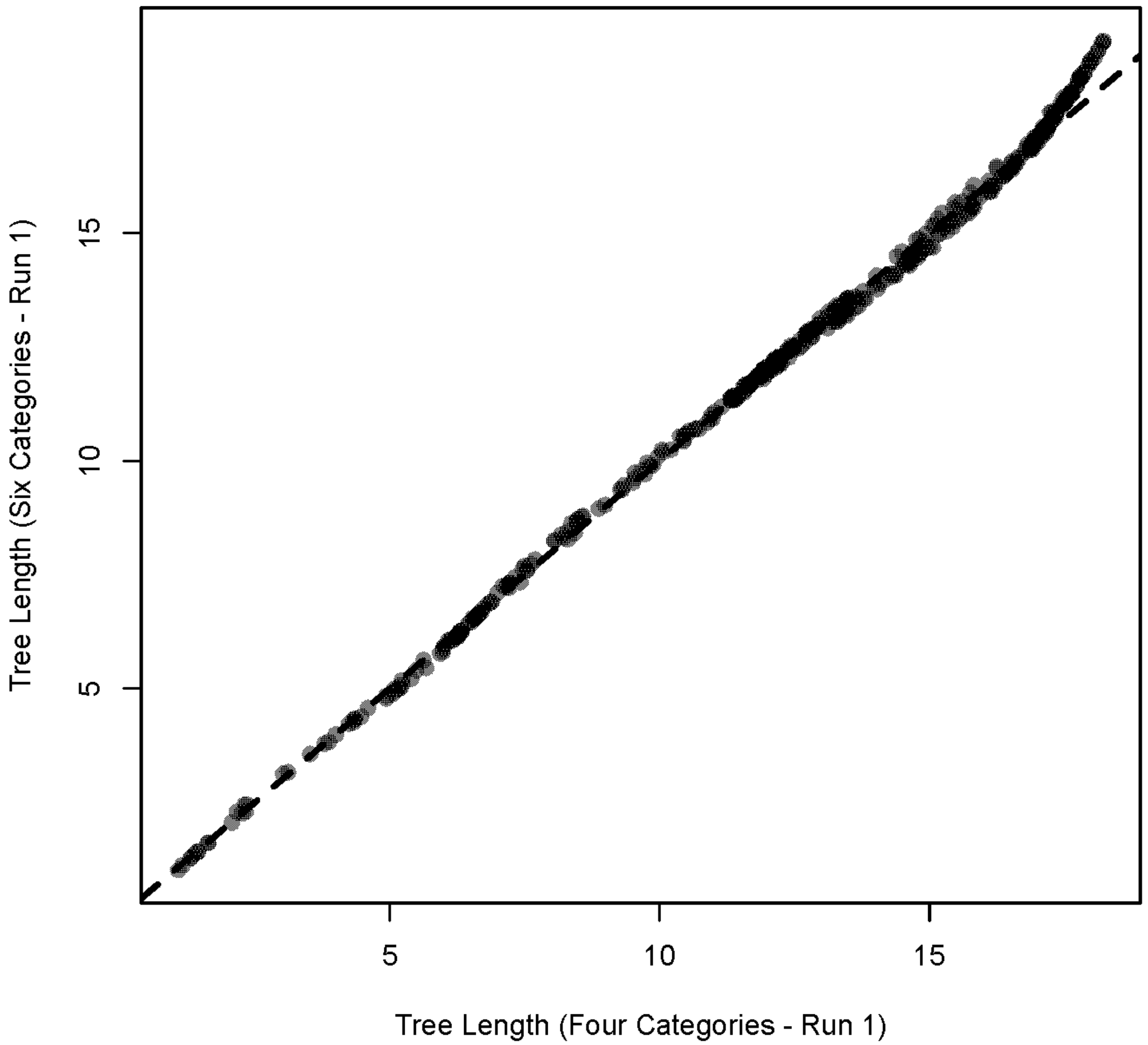

Figure S3. A comparison of site-rate (tree length) estimates between FMMs with four and six categories. Each point represents one site in the alignment of all gK domains. The mean posterior tree length from the four-category FMM is given on the *x*-axis and the corresponding value from the six-category FMM is given on the *y*-axis. Note the overall correspondence in rates, with a slight inflection at high rates.

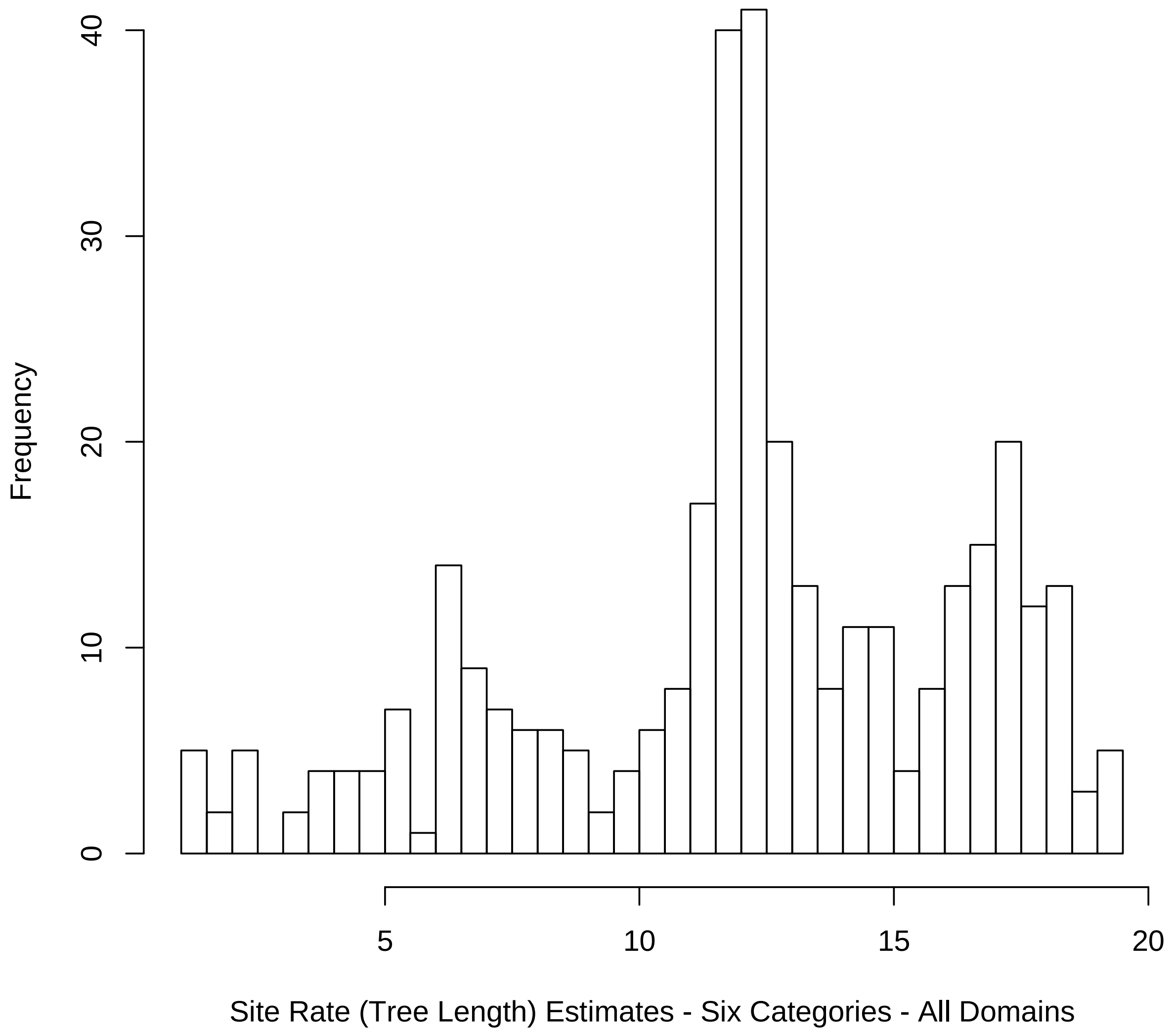

Figure S4. A histogram of site-rate (tree length) estimates for the six-category FMM. Tree lengths are shown for all 478 sites in the gK alignment, using the mean posterior tree length for each site.

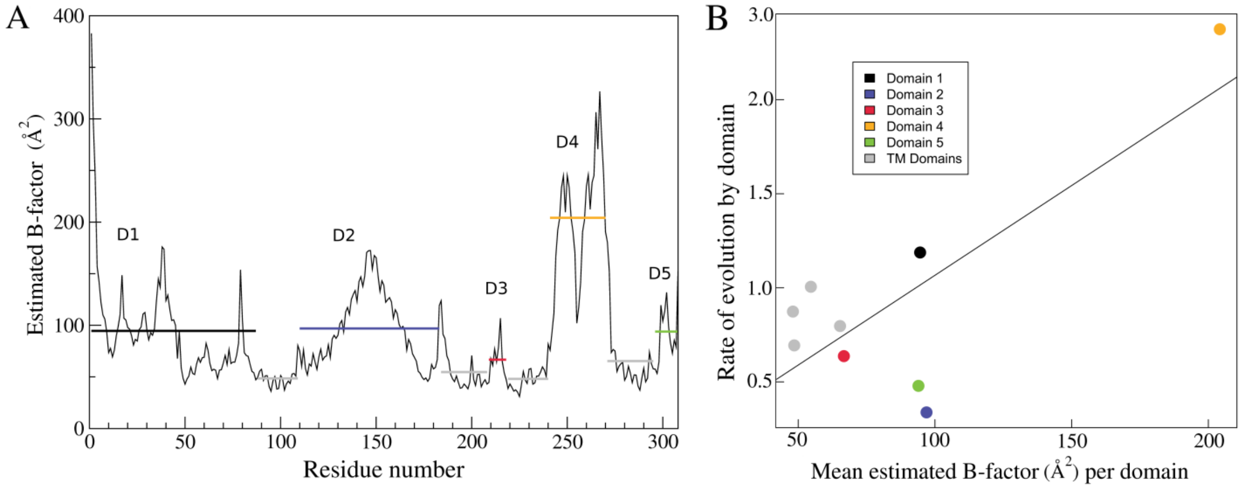

Figure S5. A domain-specific comparison of Root Means Sqare Fluctuation (RMSF) and relative evolutionary rate. A. RMSF are estimated based on Gaussian Network Model (GNM), which is a form of Elastic Network Models (ENM), by ProDy python package [1]. Color lines indicate RMSF for each domain. B. Relative rates of evolution are rate multiplier values taken from fixed-partition analyses (see main text for details) and are shown on the y-axis and the mean RMSF for domains are given on the x-axis. Note the outlying position of domain 4 both in terms of RMSF and rate of evolution. Line is a best-fit for the least-squares regression of rate as a function of RMSF. We provide this line only as a heuristic, due to the small number of points and their uneven distribution. In GNM and ENM amino acids closer than 5 A are connected with imaginary springs to form a network and the movement of the amino acids (elasticity) is calculated[1]. The elasticity calculation is then used to estimate the Root Mean Square fluctuation.

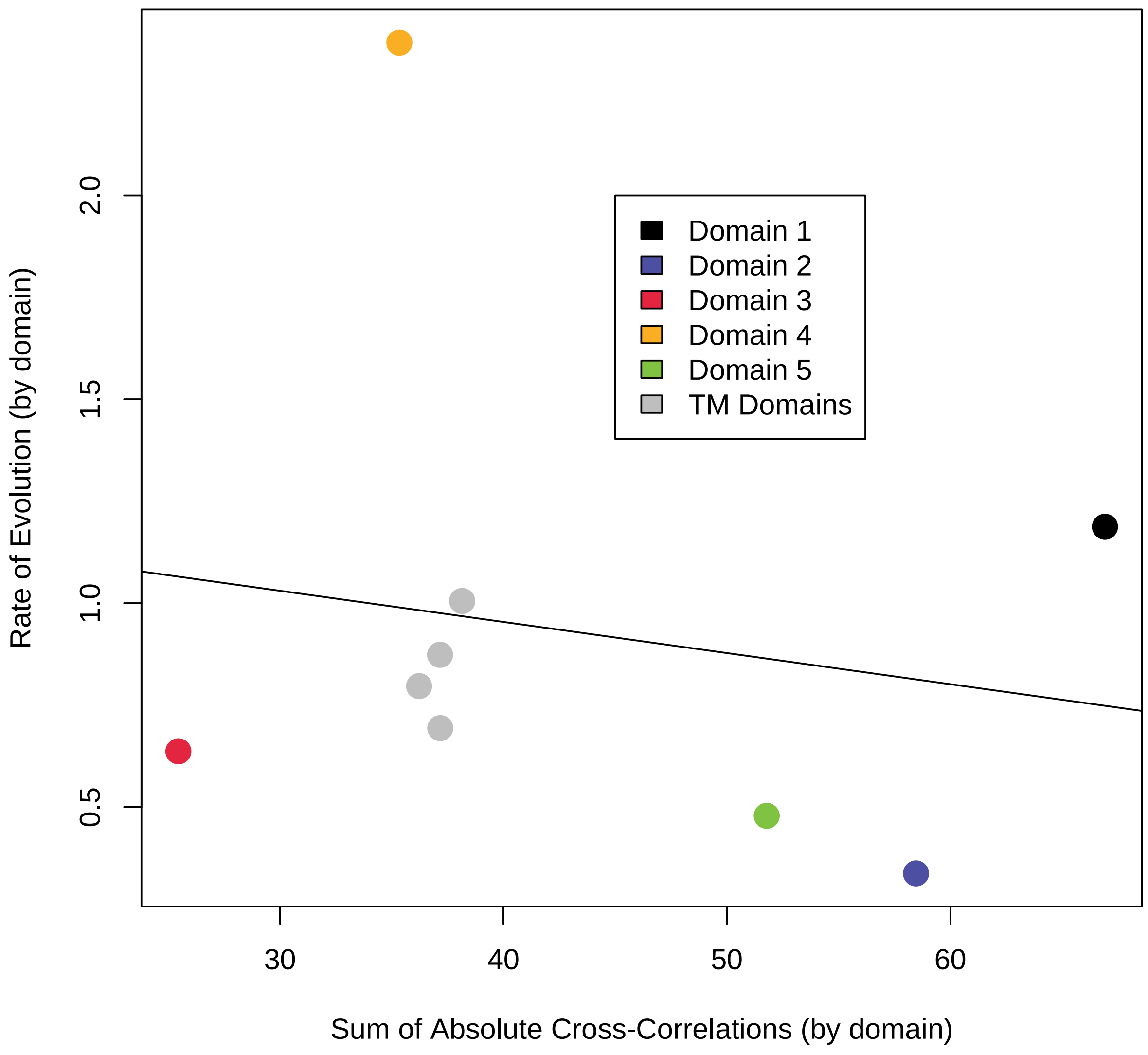

Figure S6. A domain-specific comparison of the average sum of absolute cross-correlations and relative evolutionary rate. Relative rates of evolution are rate multiplier values taken from fixed-partition analyses (see main text for details).

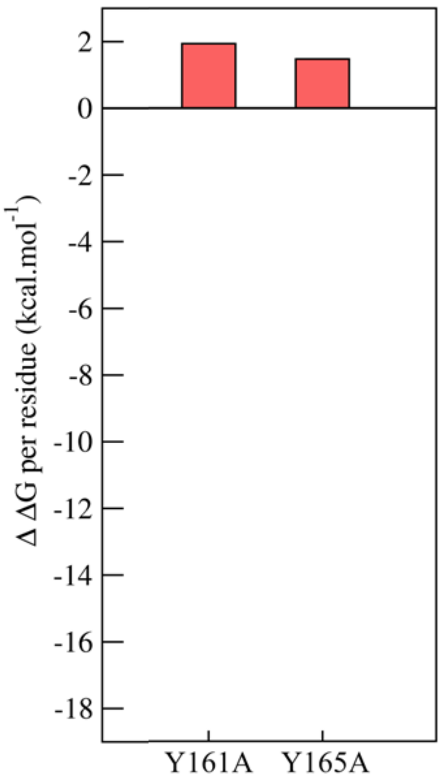

Figure S7. Calculation of change in free energy (ΔΔG) of the folded HSV-1 gK for tyrosine mutations. Using FoldX the effect of single amino acid mutations to Alanine on the free energy of the folded protein model was calculated [3].

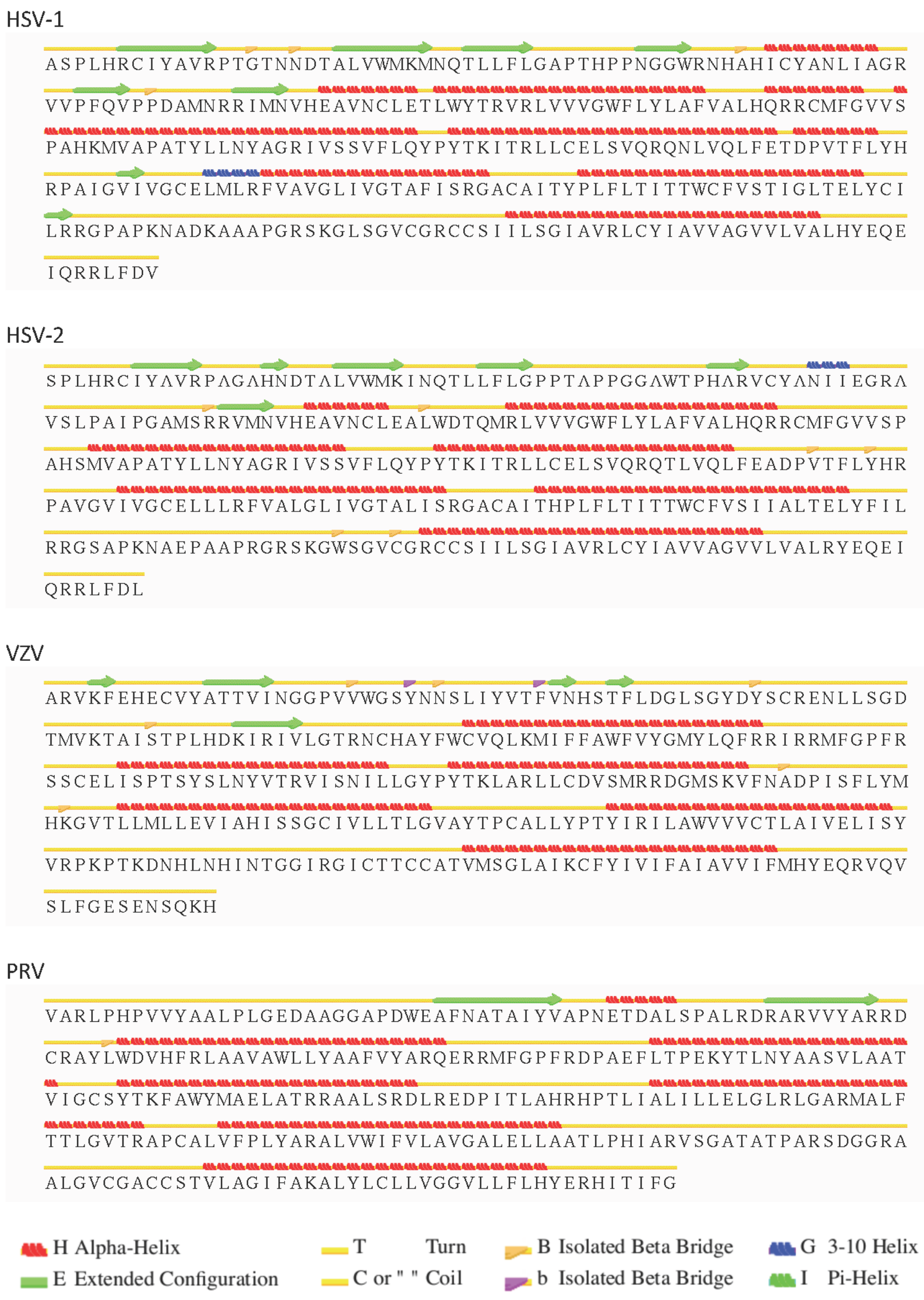

Fig.S8. Secondary structure assigned with STRIDE to structure models of gK from HSV-1, HSV-2, VZV, and PRV.

dgK	Defect in egress [4,5]<br>Defect in neuroinvasion [6]
gK 1-139(dhpd1)	Small plaque(<dgK), growth = dgK, intracellular/extracellular = dgK[7]
gK 1-239(dhpd2)	Small plaque(<dgK), growth = dgK, intracellular/extracellular = dgK[7]
gK 1-268(dhpd3)	Small plaque(>dgK), growth 5-fold greater than dgK, intracellular/extracellular = dgK[7]
gK 1-326(dhpd4)	Wild type plaque, wt growth, intracellular/extracellular = wt[7]
gK/Y183S	Small plaque(=dgK) ,growth 10-100-fold lower than dgK [7]
gK/C304S-C307S(CXXC to SXXS)	Small plaque(=dgK) [7]
gKd31-47	Small plaque, growth =dgK, intracellular/extracellular = dgK[8]
gKd31-68	Small plaque, growth 1-log reduction to wt, intracellular/extracellular < dgK[8]<br>Predominant entry by endocytosis [9]<br>Does not enter neuron via axonal termini [10]<br>Does not enter via PILRalpha [11]
gKd31-117	Small plaque, growth =dgK, intracellular/extracellular = dgK[8]
VZV ORF5 (gK) 162-340, VZV ORF5 (gK) 1-151, VZV ORF5 (gK) 1-218	No plaque[12]
dC37, dC114	dgK phenotype[13]
dC82,dC243	Syncytial [13]
N58A	Syncytial [13]
N48A	Wt [13]

T60I, L118Q, G167D	Syncytial [14]
A40V	Syncytial [15-17]
D99N, L304P, R310L	Syncytial [15]

Table S1. Phenotypes of gK mutants.

1 Bakan, A., Meireles, L. M. & Bahar, I. ProDy: protein dynamics inferred from theory and experiments. *Bioinformatics* **27**, 1575-1577 (2011).

2 Still waters. *New York Times Book Review*, 16-16.

3 Schymkowitz, J. *et al.* The FoldX web server: an online force field. *Nucleic Acids Res* **33**, W382-388 (2005).

4 Jayachandra, S., Baghian, A. & Kousoulas, K. G. Herpes simplex virus type 1 glycoprotein K is not essential for infectious virus production in actively replicating cells but is required for efficient envelopment and translocation of infectious virions from the cytoplasm to the extracellular space. *Journal of virology* **71**, 5012-5024 (1997).

5 Hutchinson, L. & Johnson, D. C. Herpes simplex virus glycoprotein K promotes egress of virus particles. *Journal of virology* **69**, 5401-5413 (1995).

6 David, A. T., Baghian, A., Foster, T. P., Chouljenko, V. N. & Kousoulas, K. G. The herpes simplex virus type 1 (HSV-1) glycoprotein K(gK) is essential for viral corneal spread and neuroinvasiveness. *Current eye research* **33**, 455-467 (2008).

7 Foster, T. P. & Kousoulas, K. G. Genetic analysis of the role of herpes simplex virus type 1 glycoprotein K in infectious virus production and egress. *Journal of virology* **73**, 8457-8468 (1999).

8 Chouljenko, V. N., Iyer, A. V., Chowdhury, S., Chouljenko, D. V. & Kousoulas, K. G. The amino terminus of herpes simplex virus type 1 glycoprotein K (gK) modulates gB-mediated virus-induced cell fusion and virion egress. *Journal of virology* **83**, 12301-12313 (2009).

9 Musarrat, F., Jambunathan, N., Rider, P. J. F., Chouljenko, V. N. & Kousoulas, K. G. The Amino Terminus of Herpes Simplex Virus 1 Glycoprotein K (gK) Is Required for gB Binding to Akt, Release of Intracellular Calcium, and Fusion of the Viral Envelope with Plasma Membranes. *Journal of virology* **92** (2018).

10 Jambunathan, N. *et al.* Deletion of a Predicted beta-Sheet Domain within the Amino Terminus of Herpes Simplex Virus Glycoprotein K Conserved among Alphaherpesviruses Prevents Virus Entry into Neuronal Axons. *Journal of virology* **90**, 2230-2239 (2015).

11 Chowdhury, S., Chouljenko, V. N., Naderi, M. & Kousoulas, K. G. The amino terminus of herpes simplex virus 1 glycoprotein K is required for virion entry via the paired immunoglobulin-like type-2 receptor alpha. *Journal of virology* **87**, 3305-3313 (2013).

12 Mo, C., Suen, J., Sommer, M. & Arvin, A. Characterization of Varicella-Zoster virus glycoprotein K (open reading frame 5) and its role in virus growth. *Journal of virology* **73**, 4197-4207 (1999).

13 Rider, P. J. F. *et al.* Cysteines and N-Glycosylation Sites Conserved among All Alphaherpesviruses Regulate Membrane Fusion in Herpes Simplex Virus 1 Infection. *Journal of virology* **91** (2017).

14 Sarfo, A. *et al.* The UL21 Tegument Protein of Herpes Simplex Virus 1 Is Differentially Required for the Syncytial Phenotype. *Journal of virology* **91** (2017).

15 Dolter, K. E., Ramaswamy, R. & Holland, T. C. Syncytial mutations in the herpes simplex virus type 1 gK (UL53) gene occur in two distinct domains. *Journal of virology* **68**, 8277-8281 (1994).
16 Debroy, C., Pederson, N. & Person, S. Nucleotide sequence of a herpes simplex virus type 1 gene that causes cell fusion. *Virology* **145**, 36-48 (1985).
17 Pogue-Geile, K. L. & Spear, P. G. The single base pair substitution responsible for the Syn phenotype of herpes simplex virus type 1, strain MP. *Virology* **157**, 67-74 (1987).
